# Silver incorporated into *g*-C_3_N_4_/Alginate as an efficient and heterogeneous catalyst for promoting click and A^3^ and KA^2^ coupling reaction

**DOI:** 10.1038/s41598-021-93239-z

**Published:** 2021-07-08

**Authors:** Mansoureh Daraie, Majid M. Heravi, Pourya Mohammadi, Ali Daraie

**Affiliations:** 1 Department of Chemistry, School of Physics and Chemistry, Alzahra University, Tehran, Iran; 2 Faculty of Electrical Engineering and Robotic, Shahrood University of Technology, Shahrood, Iran

**Keywords:** Green chemistry, Heterogeneous catalysis

## Abstract

Fe_3_O_4_/*g*-C_3_N_4_/Alginate-Ag nanocomposite as a novel and effective nanocatalyst was successfully prepared. This nanocomposite was fully characterized using several techniques such as X‐ray diffraction (XRD), field emission scanning electron microscopy with energy dispersive spectroscopy (FESEM-EDS), transmission electron microscopy (TEM), and Fourier transform infrared spectroscopy (FTIR). In addition, the catalytic activity of this novel and characterized nanocatalyst was investigated in the regioselective synthesis of 1,4-disubstituted 1,2,3-triazoles via click reaction and A^3^ and KA^2^ coupling reaction in aqueous media. The prepared nanocatalyst was simply recovered by using an external magnet and reused for several times with a slight loss of catalytic activity.

## Introduction

Crucial aim of catalytic reactions in organic synthesis is the success in the selective production of target compounds with high efficiency in terms of atom economy and the yield of each reaction step. The ease of separation and recyclability of the catalyst are other noticeable challenges in this field. Hence, researchers try to design new catalysts and examine them in organic reactions to achieve new methodologies^1^. They wish to produce the products by these catalyst via green and eco-friendly steps in which minimum amount of toxic reagents or wastes is used or produced, respectively, and the needed energy and the number of reaction steps are reduced. Currently, one-pot reactions and heterogeneous catalysis are the most attractive choices to design new methods for the synthesis of target compounds because this way saves the consuming energy, while separation and purification of the product is simple with no need to isolate the intermediates, and the catalyst is filtered and possibly reused^2^. Besides, the structure of heterogeneous catalysts can be modified with various functions, otherwise diverse functions in a catalyst can work separately or cooperatively in different steps^3,4^.

Incorporation of metal nanoparticles (NPs) onto a substrate of catalyst has received increased attention in recent years. This is as a result of improvements in catalytic methodologies, particularly by the development of bottom-up approaches. Preparation and stabilization of metal NPs need specific capping organic molecules, including polymers, ligands, and surfactants, to control the NPs size and to prevent agglomeration of particles^5^.

The ways for the preparation of distinct supported metal NPs and innovation of procedures for the isolation and recycling of catalysts are of hot topics in catalytic research. In this regard, researchers have drawn their attention on the design of new supported magnetic NPs based catalysts that can be effortlessly separated from the mixture using magnets^6^.

Lately, magnetic nanoparticles have been applied as excellent supports with a great surface-to-volume ratio led to notable stability, great catalyst loading capability, uniform dispersion, and suitable recycling^7^.

Silver nanoparticles owing to their individual physical and chemical properties are widely used in different fields such as medicine, health, agriculture, animal husbandry, household, electronics, and packaging^8^. One of the principal problems of the usage of the nanoparticles in reactions is related to their aggregation. Stabilization of nanoparticles on proper supports overcomes the problems associated with their stability, separation, and recovery. In this regard, various supports have been utilized for the stabilization of nanoparticles such as zeolite, TiO_2_, graphene oxide, Fe_3_O_4_^9–12^, and carbon-based supports^13–17^, Among the different supports, *g*-C_3_N_4_ has shown good chemical resistance^18–20^.

1,2,3-Triazole bearing *N*-heterocyclic systems have been used in various fields of chemistry^21–24^. Even though such structures are not found in natural sources, they are acted as amide-bond surrogates in biologically active compounds owing to their large dipole moment, forming hydrogen bonds, and incredible metabolic stabilities during enzymatic degradation^25–27^.

The amide-triazole isosteric substitution in 1,2,3-triazole compounds was employed for the synthesis of a wide range of medicinal frameworks having anti-HIV, antibacterial and anticancer activities^28–32^.

In 2001, Sharpless and Meldal discovered that Copper (I) can regioselectively catalyze alkyne-azide cycloaddition and named briefly it as CuAAC reaction. This reaction was then categorized as the ‘‘paradigm’’ of all ‘‘click reactions’’^33–35^.

In order to develop the catalytic synthesis of 1,4-disubstituted 1,2,3-triazoles, some transition metals, including zinc (Zn)^36,37^, gold (Au)^38^, and nickel (Ni)^39^, were also utilized^40^. In 2013, Erick Cuevas demonstrated that AgCl complex catalyzed the formation of 1,2,3-triazoles^41^.

One type of the KA^2^ and A^3^ coupling reactions is the treatment of alkynes with amines and carbonyl compounds. Such coupling reactions are important due to the construction of propargylamines as valuable substrate in the architecture of various organic compounds, including natural products, bioactive nitrogen-rich compounds such as fungicides and herbicides, and heterocyclic structures such as quinolines, pyrroles, and indolizines^42–46^. Remarkably, KA^2^ and A^3^ coupling reactions not only are employed in the synthesis of propargylamines, but also are considered as alternatives for the classic synthesis of propargylictriflates and propargylic phosphates. Transition metal based catalysts including Au, Zn, Cu, Ag, and Fe can promote such coupling reactions^47,48^.

Because of the importance of coupling reactions in art of synthesis, recently researches has been focused on the deletion of their drawbacks, especially by designing nontoxic heterogeneous catalysis^49–53^.

In attempt to disclose the utility of C_3_N_4_ as a promising catalyst support for design and synthesis of heterogeneous catalysts^54–57^, in this work, we claim the preparation and full characterization of Fe_3_O_4_/g-C_3_N_4_/Alginate-Ag nanocomposite as an effective and novel nanocatalyst in the regioselective synthesis of 1,4-disubstituted -1,2,3-triazoles via Click^58–60^ and A^3^ and KA^2^^61^ coupling reactions under mild and environmentally benign conditions.

## Experimental

### Materials and instruments

The catalyst was synthesized using the following chemical materials: Thiourea, Polyalginate, AgNO_3_, NH_3_, FeCl_3_·6H_2_O, FeCl_2_·4H_2_O and hydrazine hydrate. All purchased from Sigma-Aldrich and used without any purification.

For the synthesis of triazole derivatives and A^3^ and KA^2^ coupling products, α-haloketones or alkyl halides, terminal alkynes and sodium azide, morpholine or piperidine and different aromatic aldehyde were used. These compounds were obtained from Sigma-Aldrich on analytical grade. The progress of click reaction was monitored by TLC silica gel 60 F254, using ultraviolet light.

To characterize Fe_3_O_4_-g-C_3_N_4_-Alginate-Ag, SEM, EDX, XRD, TEM, FTIR, and ICP-AES were employed. FTIR spectra of each hybrid component of catalyst was recorded using KBr disks on FTIR spectrometer Bruker Tensor 27 in the 400–4000 cm^−1^ region. SEM image of the catalyst was obtained from a FESEM-TESCAN-MIRA3 microscope coupled with EDX (TSCAN). X-ray diffraction (XRD) pattern was achieved by a Co Kα radiation (λ = 1.78897 Å, 40 keV and 40 Ma).

### Synthesis of Fe_3_O_4_-g-C_3_N_4_-Alginate-Ag magnetic nanocatalyst

#### Synthesis of g-S-C_3_N_4_

Thiourea (5.0 g) was put into a crucible and then heated at 550 °C and for 3 h. Then, the product (g-C_3_N_4_) was powdered.

#### Synthesis of Fe_3_O_4_-g-C_3_N_4_

At first, 1.0 g of g-C_3_N_4_ was added to the 120 mL of H_2_O and then was dispersed, after that, Fe^3+^and Fe^2+^ with molar ratio 2:1 were added to the reaction mixture. Then, it was heated at 55 °C. Next, 10 mL of ammonia solution (28%) was poured to it. This mixture was stirred for 1 h. After the end of reaction, the precipitated was separated by an external magnet, and washed several times with distilled water and ethanol (2:1) and dried at 25 °C for overnight.

#### Synthesis of Fe_3_O_4_-g-C_3_N_4_-Alg-Ag

**Figure 1:**
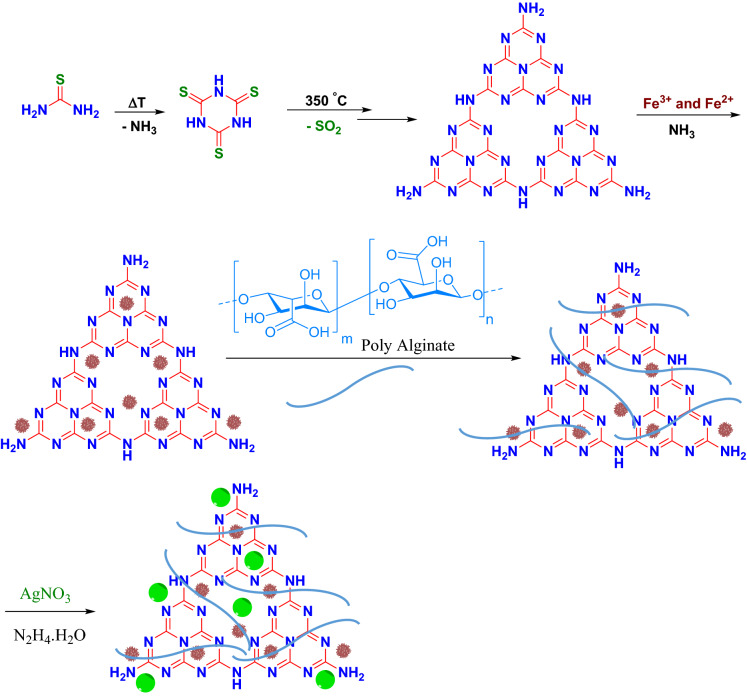
The schematic route for the preparation of Fe_3_O_4_-g-C_3_N_4_-Alg-Ag catalyst.

Typically, 1.0 g of Fe_3_O_4_-*g*-C_3_N_4_ was poured in distilled water (40 mL) and stirred for 30 min. Then 20 mL of the solution of the alginate polymer (1.5%) was added to the above of the reaction mixture. The mixture was stirred at room temperature for 5 h. Then, 30 mL of the AgNO_3_ (3 mM) was added. This mixture was stirred for 2 h, after that 0.5 mL diluted hydrazine hydrate was poured and stirred for 24 h. Finally, the magnetic precipitate was collected with an external magnet. The target product washed with distilled water and ethanol (2:1) and dried at 25 °C for overnight. The ICP-AAS analysis was used to the determination of Ag immobilized on the prepared nanocatalyst, that the results showed, 0.9 mmol of Ag loaded in the 1 g of catalyst (Fig. 1).

### General procedure for the synthesis of 1,4-disubstituded 1,2,3-triazoles

**Figure 2:**
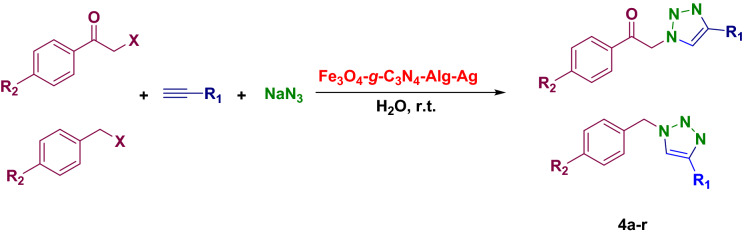
Synthesis of 1,4-disubstituted 1,2,3-triazoles.

To a mixture of alkyl halide or α-haloketone (1 mmol), sodium azide (1.2 mmol), and terminal alkyne (1 mmol) in water (5 ml), Fe_3_O_4_-*g*-C_3_N_4_-Alg-Ag (0.02 g) as a catalyst was added and the resulting mixture was magnetically stirred for the appropriate time. At the end of the reaction (monitored by TLC), the solid was filtered off and recrystallized in EtOH (Fig. 2).

### Typical procedure of A^3^ and KA^2^ coupling reaction

**Figure 3:**
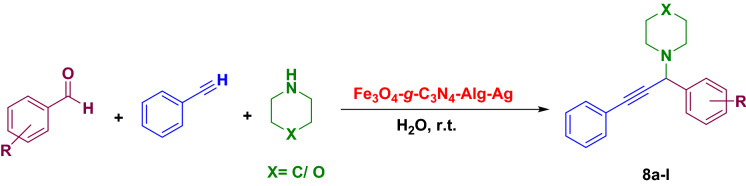
A3 and KA2 coupling reactions.

0.02 g of Fe_3_O_4_-*g*-C_3_N_4_-Alg-Ag was poured into a round bottom flask containing an aqueous mixture of alkyne (1.1 mmol), aromatic aldehyde (1 mmol), and piperidine/morpholine (1 mmol). After stirring and heating the reaction mixture for appropriate time, which was monitored by TLC, it was cooled down and filtered (Fig. 3). The crude product comprising catalyst was dissolved in hot EtOH and the residue catalyst was filtered. Then, the filtrated was cooled to give the crystalized pure product.

## Result and discussion

### Catalyst characterization

**Figure 4:**
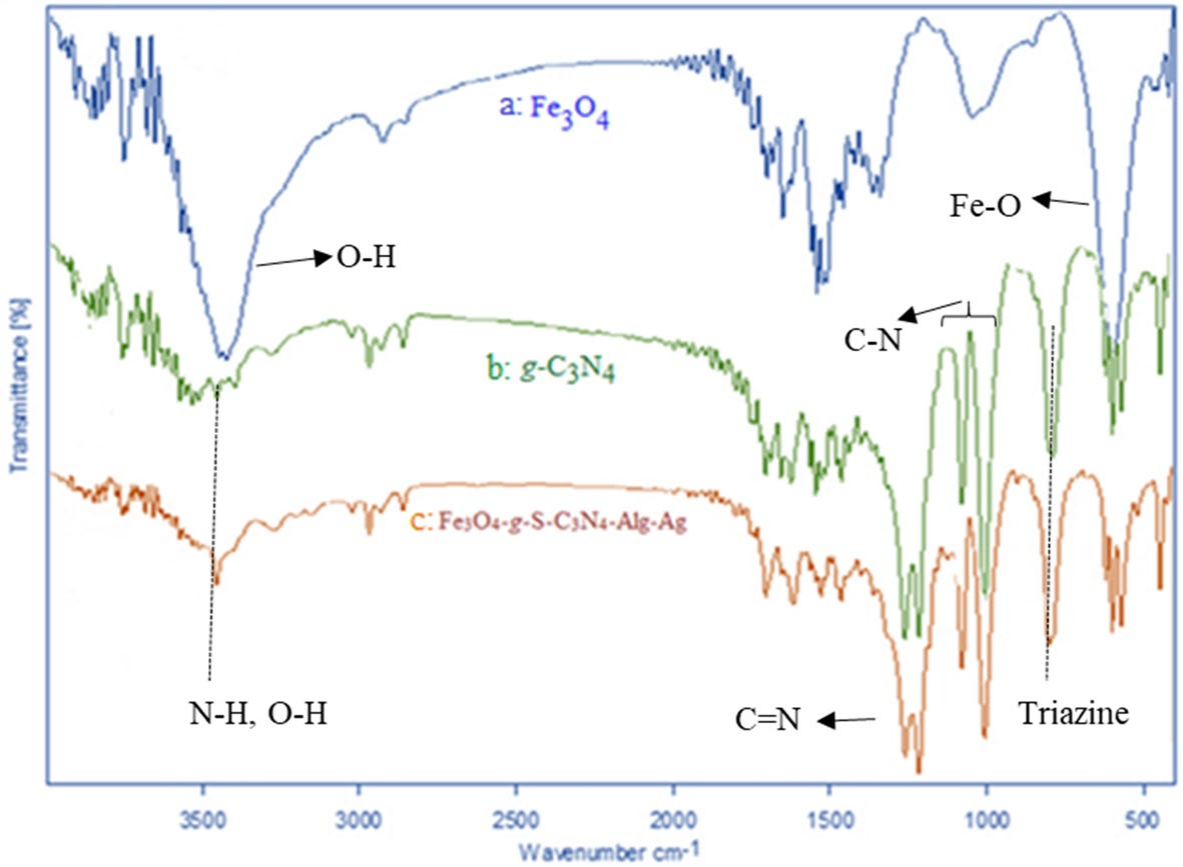
FT-IR spectra of (**a**) Fe_3_O_4_, (**b**) g-C_3_N_4_ and (**c**) Fe_3_O_4_-g-C_3_N_4_-Alg-Ag nanomaterials.

FT-IR spectra of Fe_3_O_4_-*g*-C_3_N_4_-Alg-Ag nanocatalyst was recorded to detect the functional groups in this catalyst. As shown in Fig. 4a, FT-IR spectrum of Fe_3_O_4_ displays a strong absorption band at 597 cm^−1^ belonging to the tetrahedral structure of trivalent Fe–O absorption. The hydroxyl groups existing in the Fe_3_O_4_ surface illustrates a broad band at 3422–3500 cm^−1^. Figure 4b shows a strong band at 797 cm^−1^ belonging to the special bending vibration of triazine moiety. The bands appeared at the range of 1200 to 1400 cm^−1^ are related to the stretching vibration of C–N group. The peak appeared at 1611 cm^−1^ is due to the stretching vibration of C=N group. In addition, stretching vibration of NH has appeared at 3450 cm^−1^. FT-IR spectrum of Fe_3_O_4_-*g*-C_3_N_4_-Alg-Ag nanostructure is shown in Fig. 4c. As Fe_3_O_4_-*g*-C_3_N_4_ was covered with the polymer, the weak band at 2963 cm^−1^ probably is due to the stretching vibration of aliphatic CH in the polymer structure. Figure 4c show the FT-IR spectrum of Fe_3_O_4_-*g*-C_3_N_4_-Alg-Ag in which there is no change by comparing it with its substrate. Fe_3_O_4_-*g*-C_3_N_4_-Alg-Ag is formed after decomposition of Ag NPs on the Fe_3_O_4_-*g*-C_3_N_4_-Alg, and this spectrum shows the stability of Fe_3_O_4_-*g*-C_3_N_4_-Alg during synthesis of Ag NPs.

**Figure 5:**
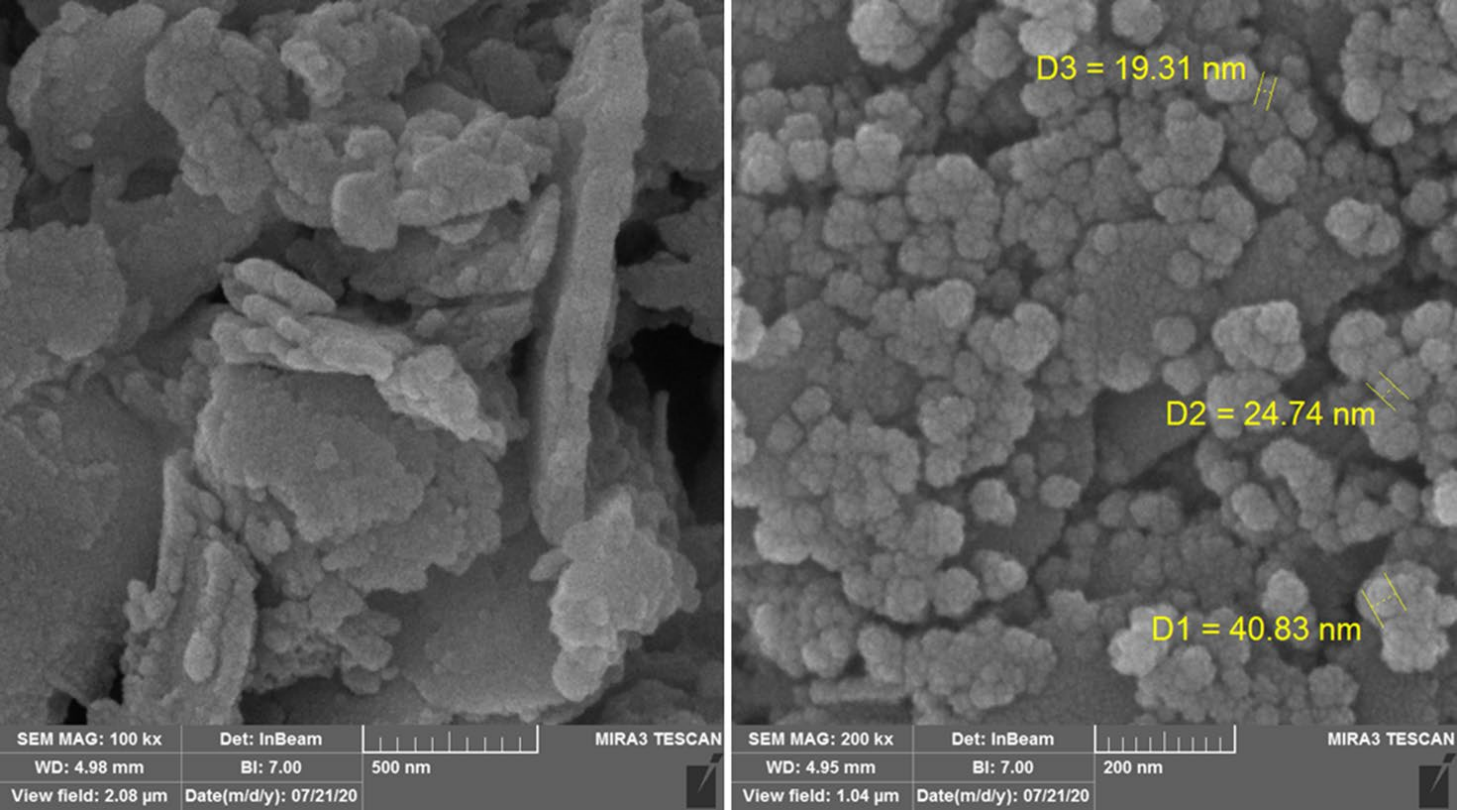
SEM image of Fe_3_O_4_-g-C_3_N_4_-Alg-Ag nanomaterials.

By using scanning electron microscopy (SEM) the morphology and size of the Fe_3_O_4_-*g*-S-C_3_N_4_-Alg-Ag synthesized nanocatalyst were investigated (Fig. 5). The particles almost have a spherical and the average size of them is about 28 nm. Whereas the surface of the g-C_3_N_4_ covered with Fe_3_O_4_ and Ag nanoparticles cannot be observed of theirs.

**Figure 6:**
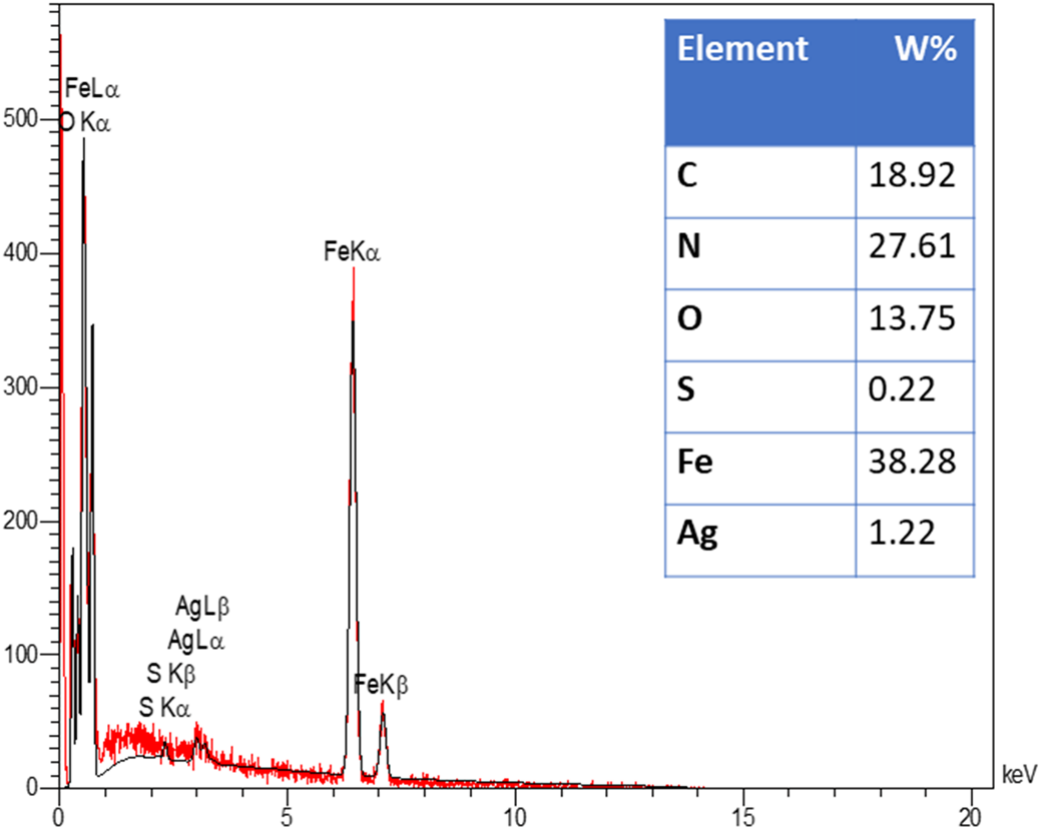
EDX analysis of Fe_3_O_4_-g-C_3_N_4_-Alg-Ag nanomaterials.

**Figure 7:**
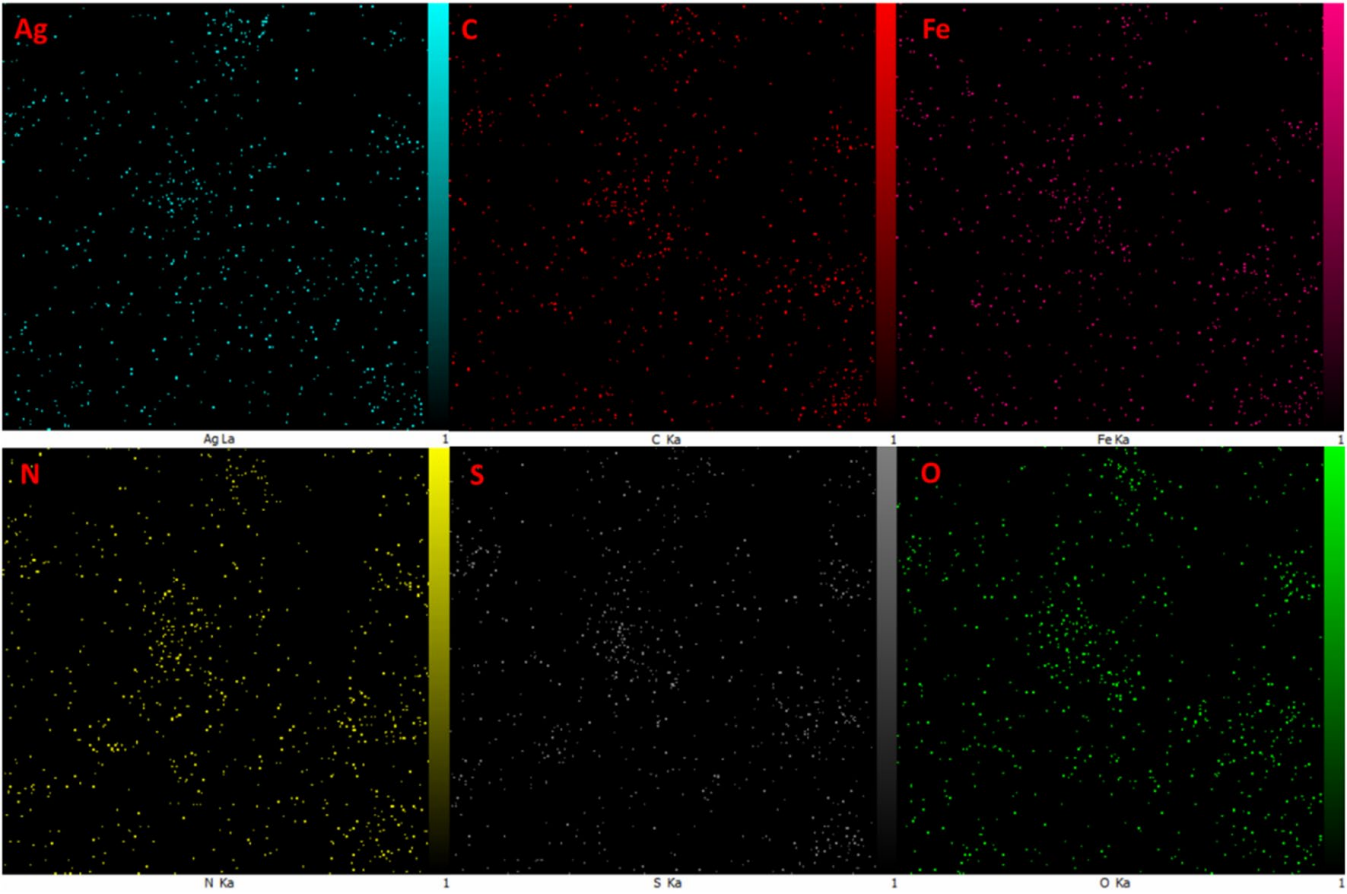
EDX-mapping study of Fe_3_O_4_-g-C_3_N_4_-Alg-Ag nanomaterials.

The EDX analysis as elemental analysis confirmed the presence of Ag, O, Fe, C, S, and N elements which can be confirmed elemental analysis of prepared catalyst (Fig. 6). The EDX-mapping images of synthesized nanocatalyst illustrated in Fig. 7. The elements of Ag, O, Fe, C, S, and N were goodly dispersed into the nanocatalyst.

**Figure 8:**
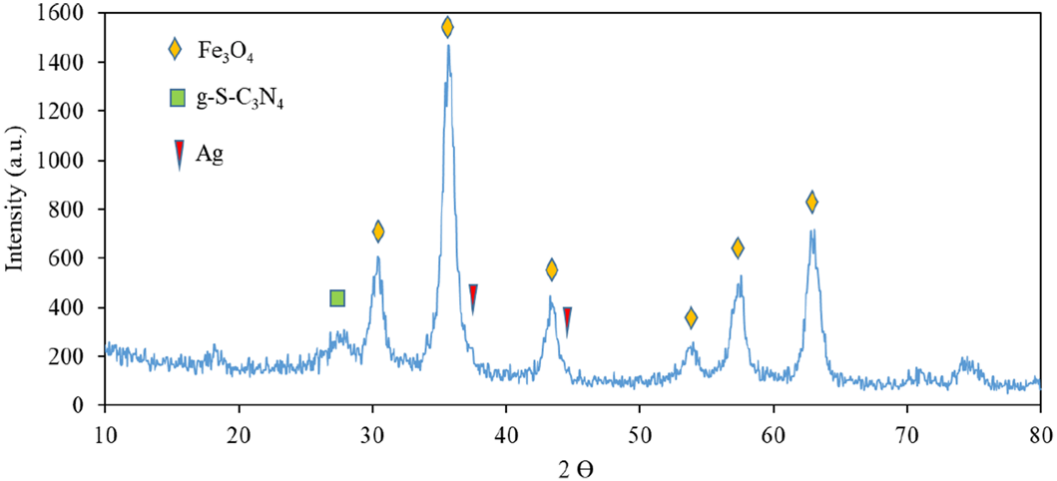
XRD pattern of Fe_3_O_4_-g-C_3_N_4_-Alg-Ag nanomaterials.

The XRD technique is as a method for considering crystal structures of the material. The XRD pattern of the Fe_3_O_4_-g-C_3_N_4_-Alg-Ag nanocatalyst magnetic was illustrated in the Fig. 8.

The recorded peaks at 2Ө = 30.1°, 35.4°, 43.1°, 53.5°, 57.2°, and 62.7°, related to the planes (2 2 0), (3 1 1), (4 0 0), (4 2 2), (5 1 1), and (4 4 0) respectively, that can be confirmed face-centered cubic structure of Fe_3_O_4_ nanoparticles (JCPDS card no. 19‐0629). Also the XRD pattern of Ag nanoparticles shows the peaks at 2θ = 38.0°, 44.2°, 64.5°, and 77.5° that can be related to the planes (1 1 1), (2 0 0), (2 2 0) and (3 1 1) respectively, (JCPDS card no. 65‐2871), the planes g-C_3_N_4_ shows a peak at 2θ = 28° (JCPDS card no. 87-1526). This peak confirmed the presence of g-C_3_N_4_.

**Figure 9:**
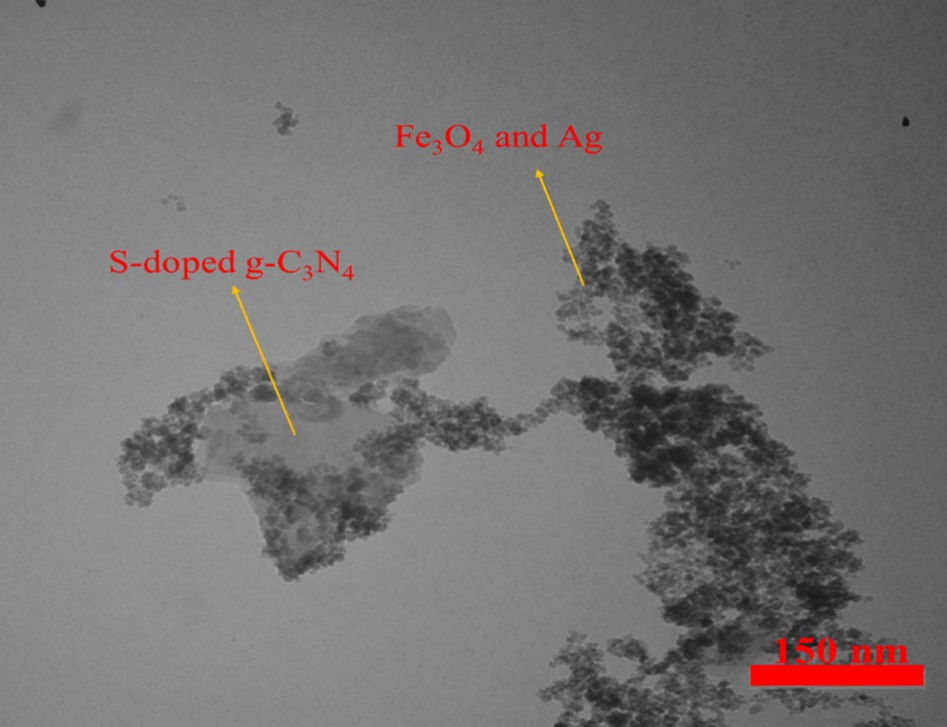
TEM image of the Fe_3_O_4_-g-C_3_N_4_-Alg-Ag nanomaterials.

According to the TEM analysis, Fe_3_O_4_ and Ag nanoparticles were dispersed on the g-C_3_N_4_-Alginate. TEM images display the mean size of the particles are about 12 nm. It also shows that g-C_3_N4 sheets are nanoscale (Fig. 9).

### Catalytic activity

It was found that this is a potent catalyst in organic transformations. To study this supposition, Fe_3_O_4_-g-C_3_N_4_-Alginate-Ag was used as catalyst in the Click reaction of sodium azide, α-haloketone or alkyl halide and terminal alkynes for the synthesis of triazoles. Primarily, the reaction of phenylacetylene, benzyl bromide and sodium azide was designated as the model reaction and run in the different solvents and also under solvent‐free conditions. Pleasantly, by comparing the yields of the model reactions in different solvents, it was confirmed that water as the best protic solvent gained the highest yield of the desired product. Afterward, to find the optimum reaction temperature and the effective amounts of catalyst, the model reaction was repeated in the presence of various catalyst amounts at different reaction temperatures (Table 1).

**Table 1:** Optimization reaction condition using Fe_3_O_4_/s-C_3_N_4_-Starch-Ag As catalyst.

Entry	Loading of catalyst (g)	Condition (temp. (ºC))	Time (min)	Yield (%)
1	0.02	H_2_O/r.t.	15	98
2	0.02	H_2_O/50	12	92
3	0.02	H_2_O/100	12	94
4	0.02	H_2_O-EtOH (1:1)/r.t.	20	89
5	0.02	EtOH/r.t.	25	85
6	0.02	CH_3_CN/r.t.	30	75
7	0.02	CH_2_Cl_2_/r.t.	40	70
8	0.02	DMF/r.t.	45	50
9	0.02	Solvent free/r.t.	20	70
10	0.02	Solvent free/80	15	82
11	None	H_2_O/r.t.	60	30
12	0.01	H_2_O/r.t.	20	90
13	0.03	H_2_O/r.t.	15	98
14	0.04	H_2_O/r.t.	15	97

Reaction condition: phenylacetylene (1 mmol), benzyl bromide (1 mmol), sodium azide (1.3 mmol), solvent (5 ml).

The results indicated that the highest yield of the model product was achieved at room temperature in the presence of 0.02 g of catalyst. The generality of the protocol was then studied. For this purpose, Various substrates with different electron densities were participated in this reaction under optimum conditions and numerous 1,2,3‐triazoles were produced (Table 2). The results proved that Fe_3_O_4_-g-C_3_N_4_-Alginate-Ag is a good candidate to catalyze the reactions of different substrates giving the corresponding 1,2,3‐triazoles in short reaction times and great yields.

**Table 2:**
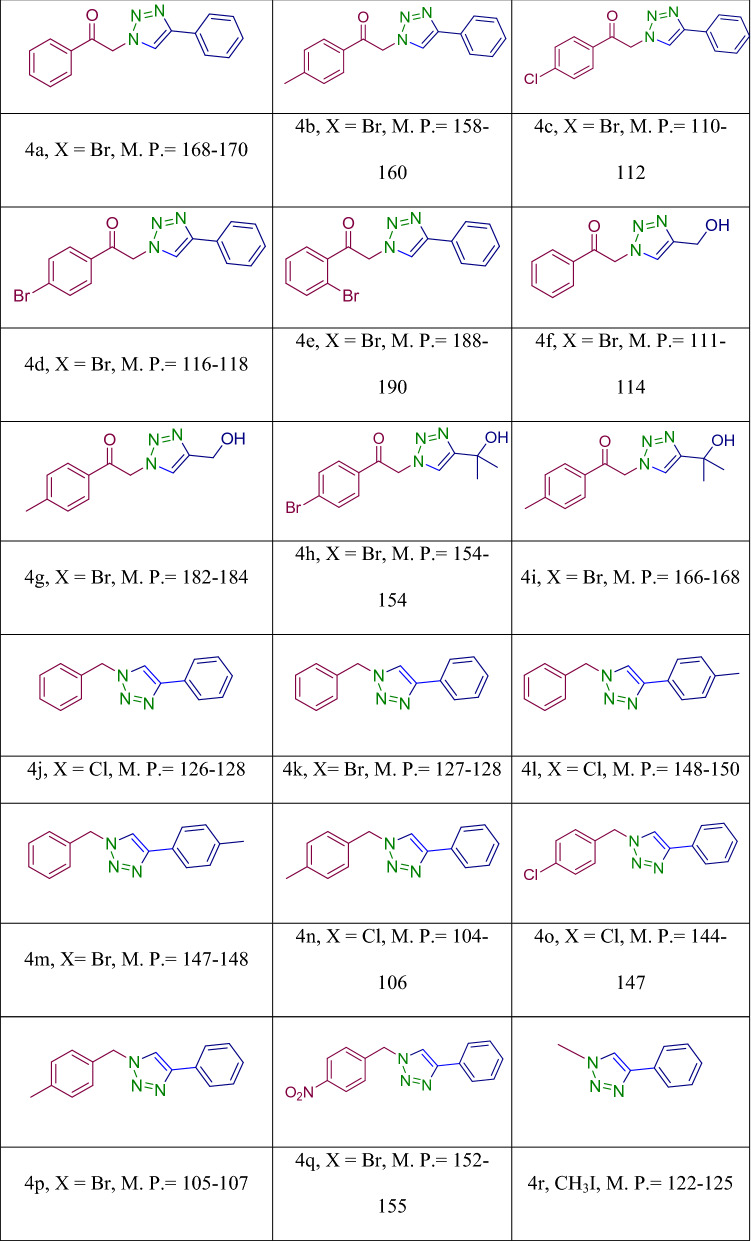
Synthesis of 1,2,3-triazoles in the presence of Fe_3_O_4_-g-C_3_N_4_-Alginate-Ag^52,53^.

### Reaction mechanism

**Figure 10:**
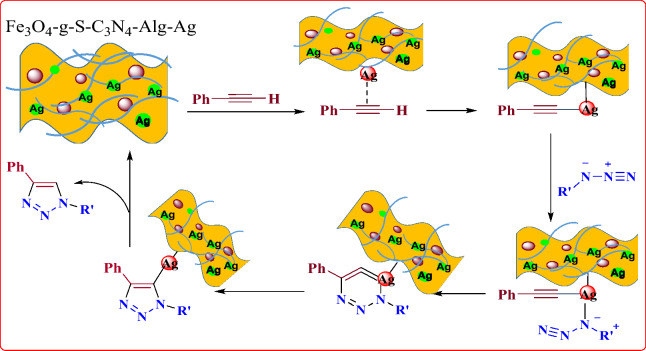
Plausible reaction mechanism.

Relied on the preceding reports^48^, a suggested probable mechanistic route for the three‐component click reaction in the presence of Fe_3_O_4_-g-C_3_N_4_-Alginate-Ag as catalyst is depicted in Fig. 10. Initially, the azide ion is served as a nucleophile group to add to benzyl halide. Instantaneously, the catalyst stimulates the terminal alkyne tolerating homocoupling reaction to attain a diyne. Lastly, the desired product 1,2,3‐triazole is provided via CuI‐mediated cycloaddition reaction.

Next, we examined the three-component A^3^ and KA^2^ coupling reactions of aromatic aldehydes, phenyl acetylene and cyclic amine in the presence of Fe_3_O_4_-g-C_3_N_4_-Alginate-Ag as a catalyst in H_2_O at ambient temperature. The coupling compounds were efficiently obtained in good to excellent yields (Table 3).

**Table 3:**
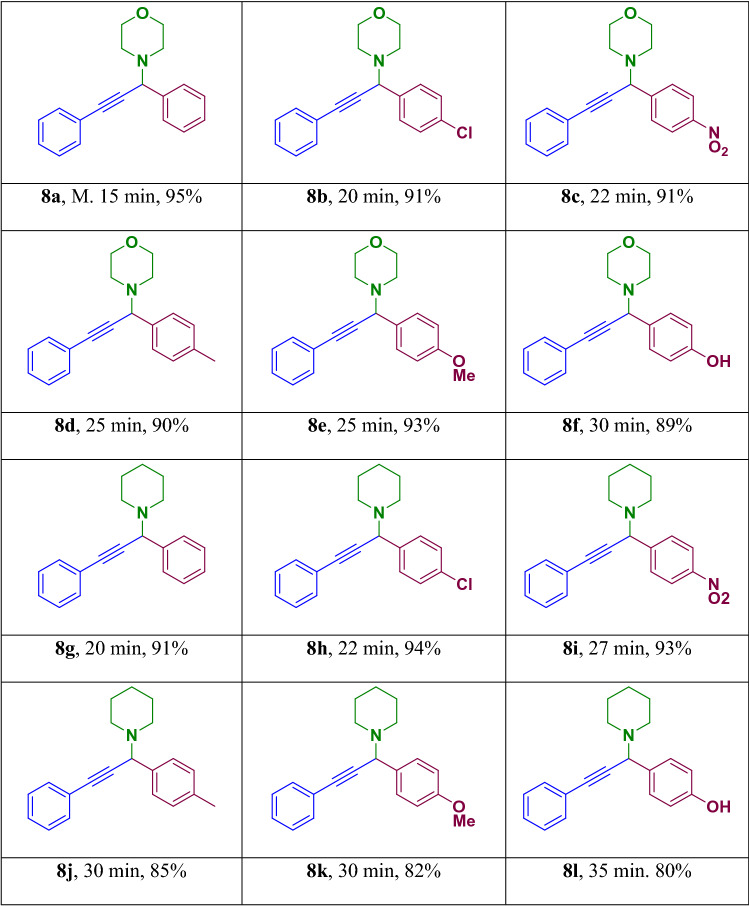
A^3^ and KA^2^ coupling reactions in the presence of Fe_3_O_4_-g- C_3_N_4_-Alginate-Ag^55,56^.

### Catalyst recyclability

**Figure 11:**
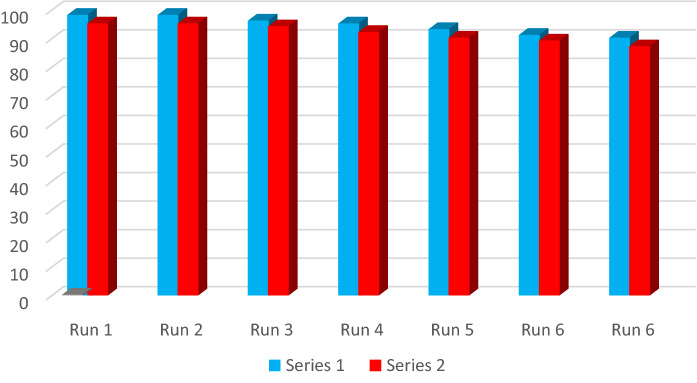
Reusability of Fe_3_O_4_-g-C_3_N_4_-Alginate-Ag.

Finally, the recyclability of Fe_3_O_4_-g-C_3_N_4_-Alginate-Ag was investigated. In this regard, the product yield in the model product was studied for 6 cycles in the presence of fresh and recycled Fe_3_O_4_-g-C_3_N_4_-Alginate-Ag (Fig. 11). The results demonstrated that Fe_3_O_4_-g-C_3_N_4_-Alginate-Ag can be recycled for seven reaction runs while its catalytic activity was not reduced.

## Conclusion

In this work, Ag nanoparticle immobilized on Fe_3_O_4_/g-C_3_N_4_/Alginate was synthesized and applied in Click and A^3^ and KA^2^ coupling reactions in water as a green solvent. The merits of these reactions are short reaction time, good efficiency and purity. The synthesized nanocatalyst also was readily separated from the reaction mixture using an external magnet, washed and reused for several runs without a significant decrease in its activity (Supplementary Information).

## Supplementary Information

Supplementary Information.
