# Supplementary material for: Silver incorporated into g-C3N4/Alginate as an efficient and heterogeneous catalyst for promoting click and A3 and KA2 coupling reaction
Source: Sci Rep. 2021 Jul 8;11:14086. doi: 10.1038/s41598-021-93239-z (PMC8266845; doi:10.1038/s41598-021-93239-z)
Supplement: Supplementary file 1 — Supplementary Information. [file 41598_2021_93239_MOESM1_ESM.docx]

**Supporting information**

**Silver incorporated into *g*-C_3_N_4_/Alginate as an efficient and heterogeneous catalyst for promoting Click and A^3^ and KA^2^ coupling reaction**

*Mansoureh Daraie^a^*, Majid M. Heravi^a^*, Pourya Mohammadi,^a^ Ali Daraie^b^*

*^a^ Department of Chemistry, School of Physics and Chemistry , Alzahra University , Tehran , Iran*

*^b^ Faculty of Electrical Engineering and Robotic, Shahrood University of Technology, Shahrood, Iran*

^*^Corresponding author: Majid M. Heravi ([m.heravi@alzahra.ac.ir](mailto:m.heravi@alzahra.ac.ir) ; [mmh1331@yahoo.com](mailto:mmh1331@yahoo.com))


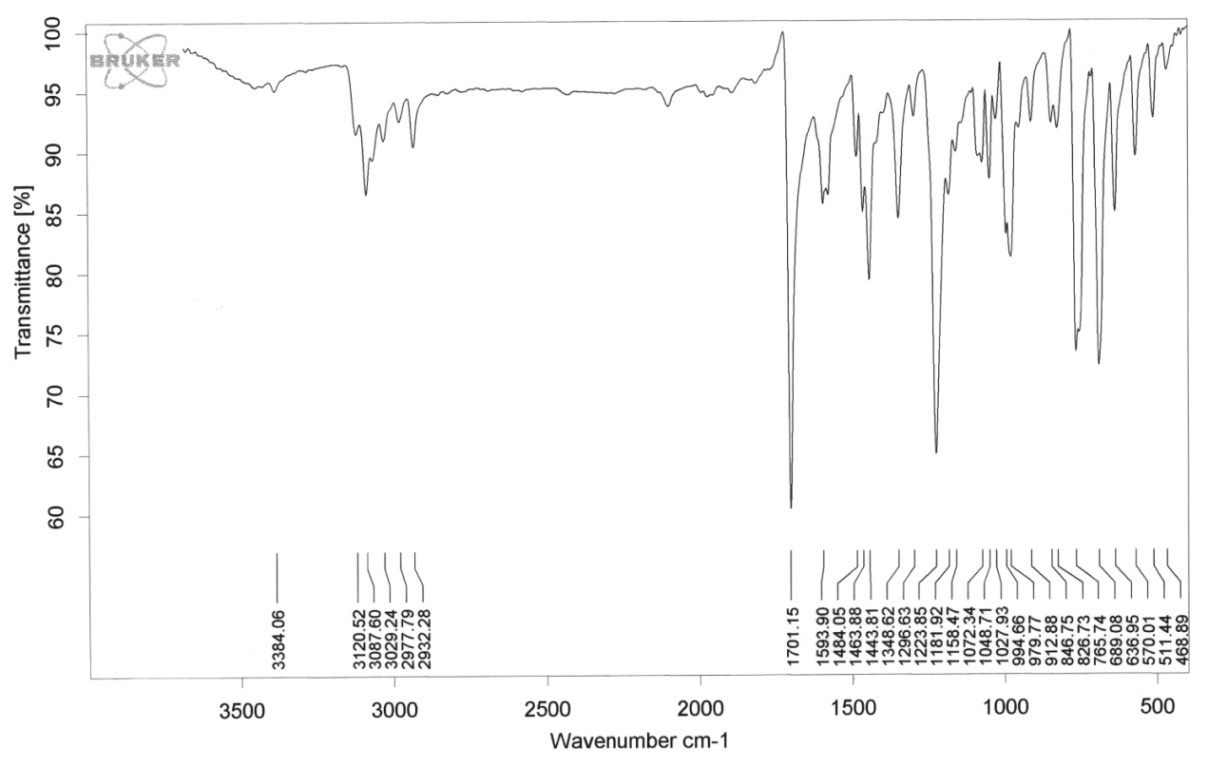


**Figure S1.** FTIR spectrum of 1-Phenyl-2-(4-phenyl-[1,2,3]triazol-1-yl)ethanone


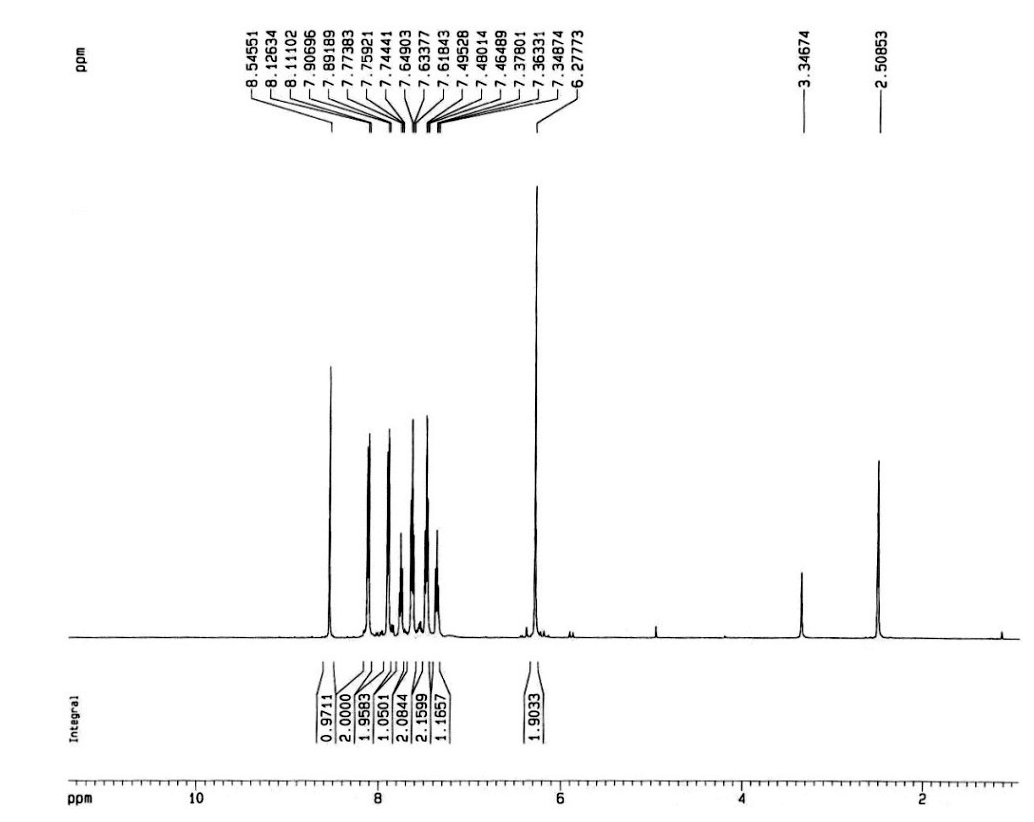


**Figure S2.** ^1^HNMR spectrum of 1-Phenyl-2-(4-phenyl-[1,2,3]triazol-1-yl)ethanone


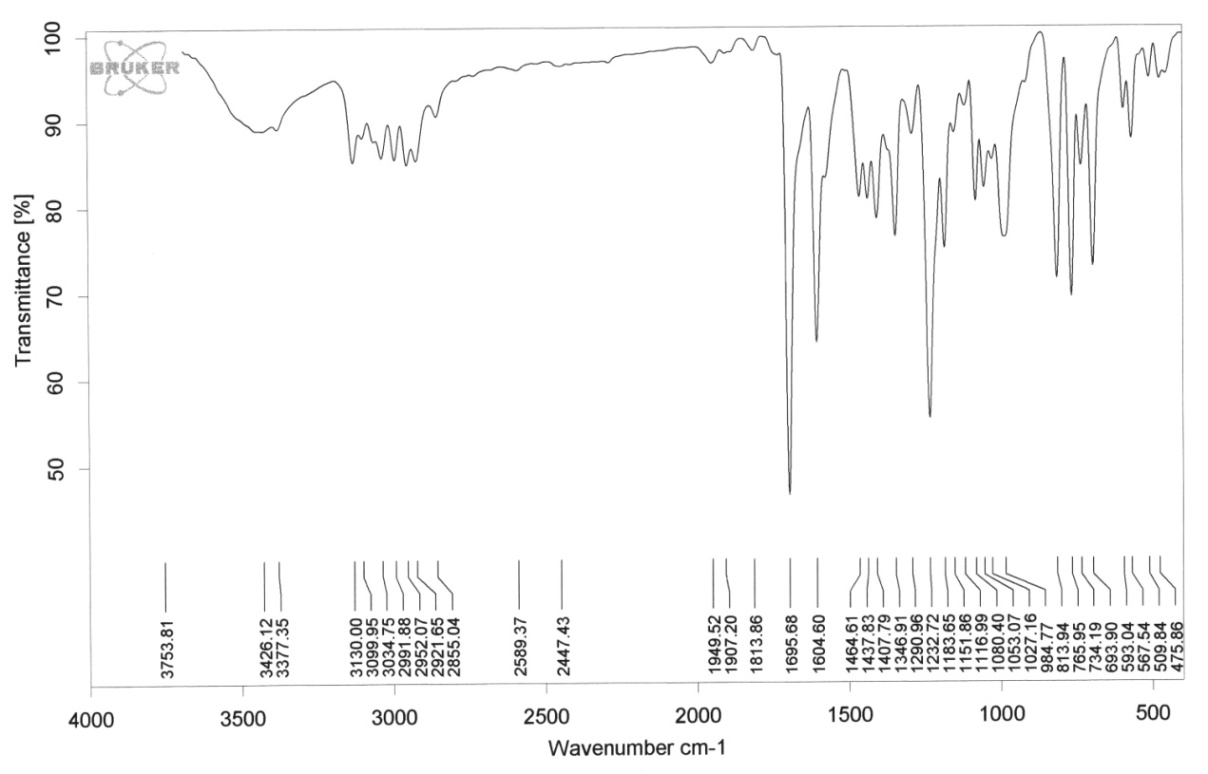


**Figure S3.** FTIR spectrum of 2-(4-Phenyl-1H-1,2,3-triazol-1-yl)-1-p-tolyl-ethanone


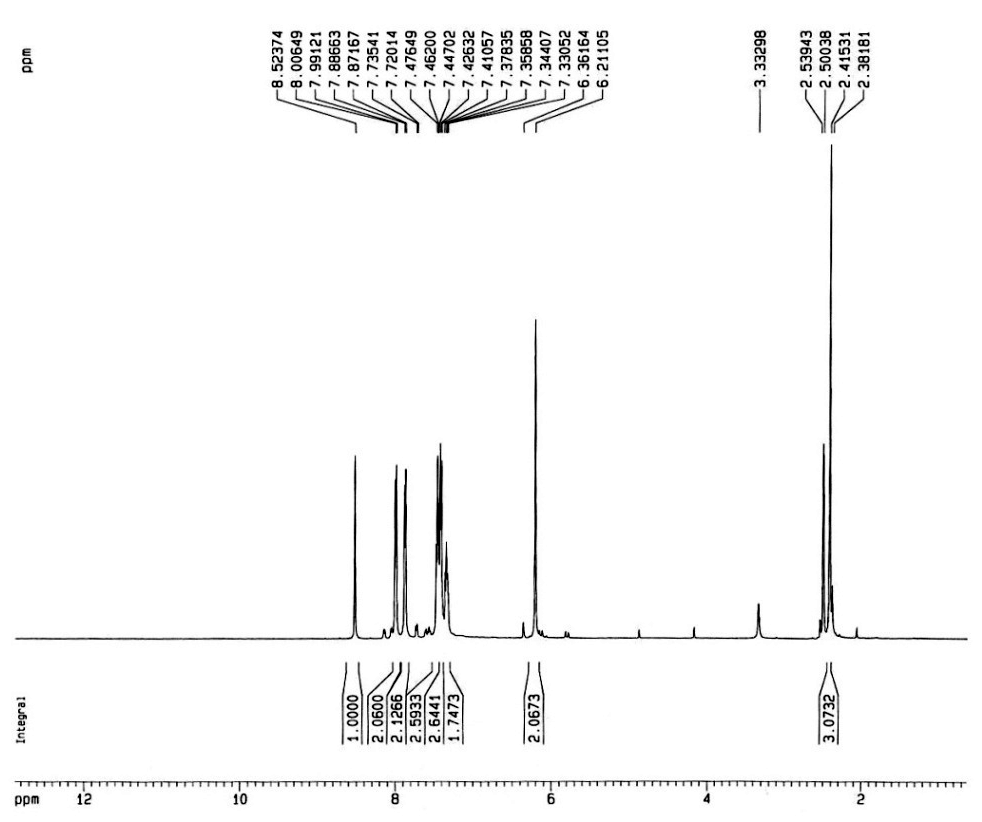


**Figure S4.** ^1^HNMR spectrum of 2-(4-Phenyl-1H-1,2,3-triazol-1-yl)-1-p-tolyl-ethanone


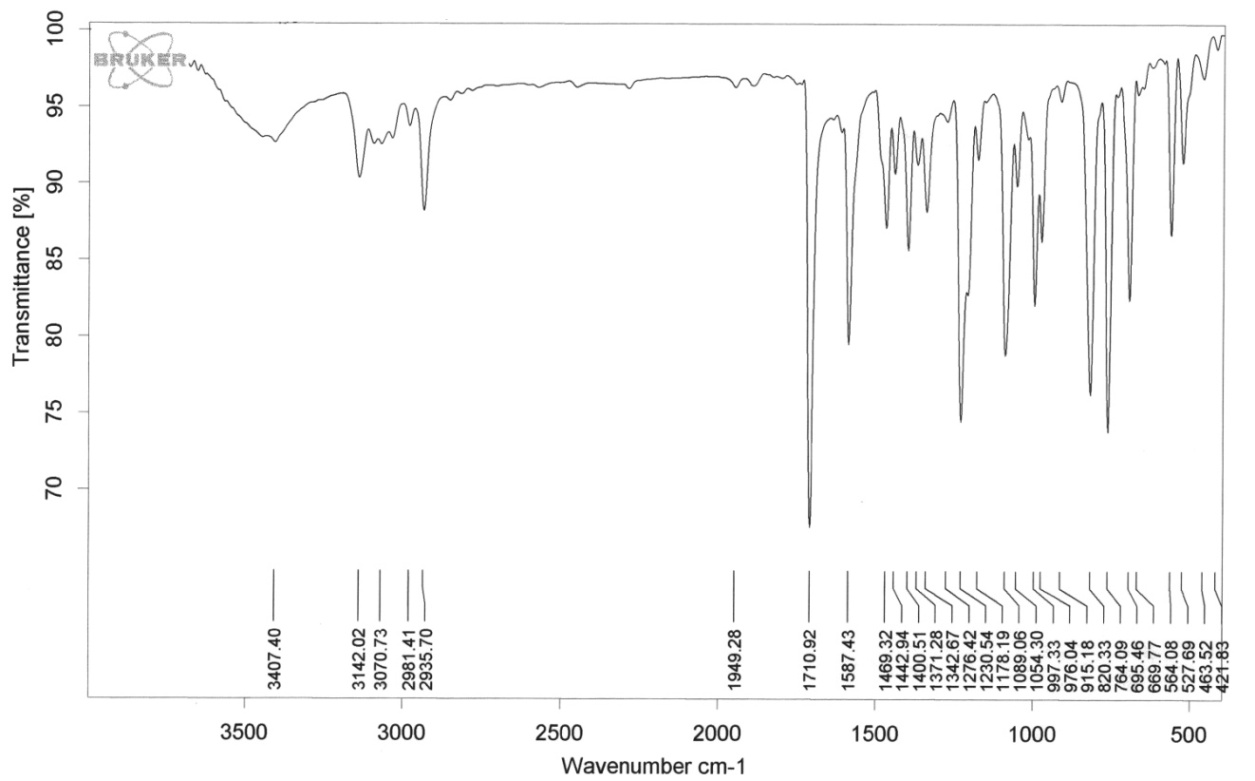


**Figure S5.** FTIR spectrum of 1-(4-Chlorophenyl)-2-(4-phenyl-1H-1,2,3-triazol-1-yl)ethanone


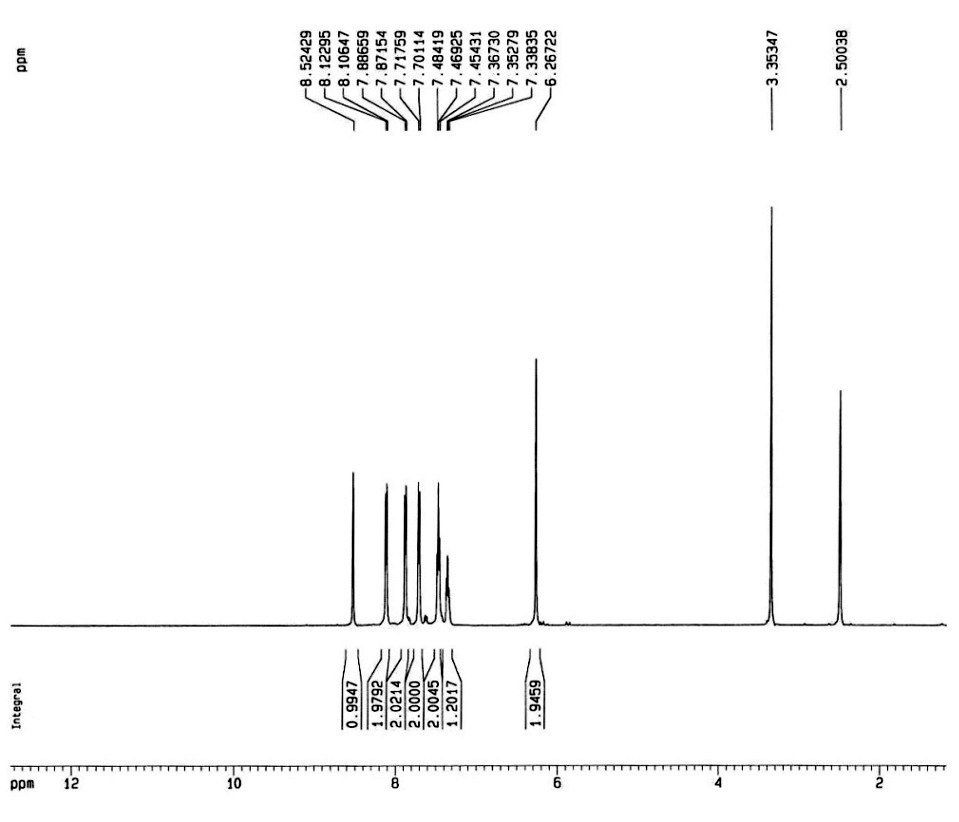


**Figure S6.** ^1^HNMR spectrum of 1-(4-Chlorophenyl)-2-(4-phenyl-1H-1,2,3-triazol-1-yl)ethanone


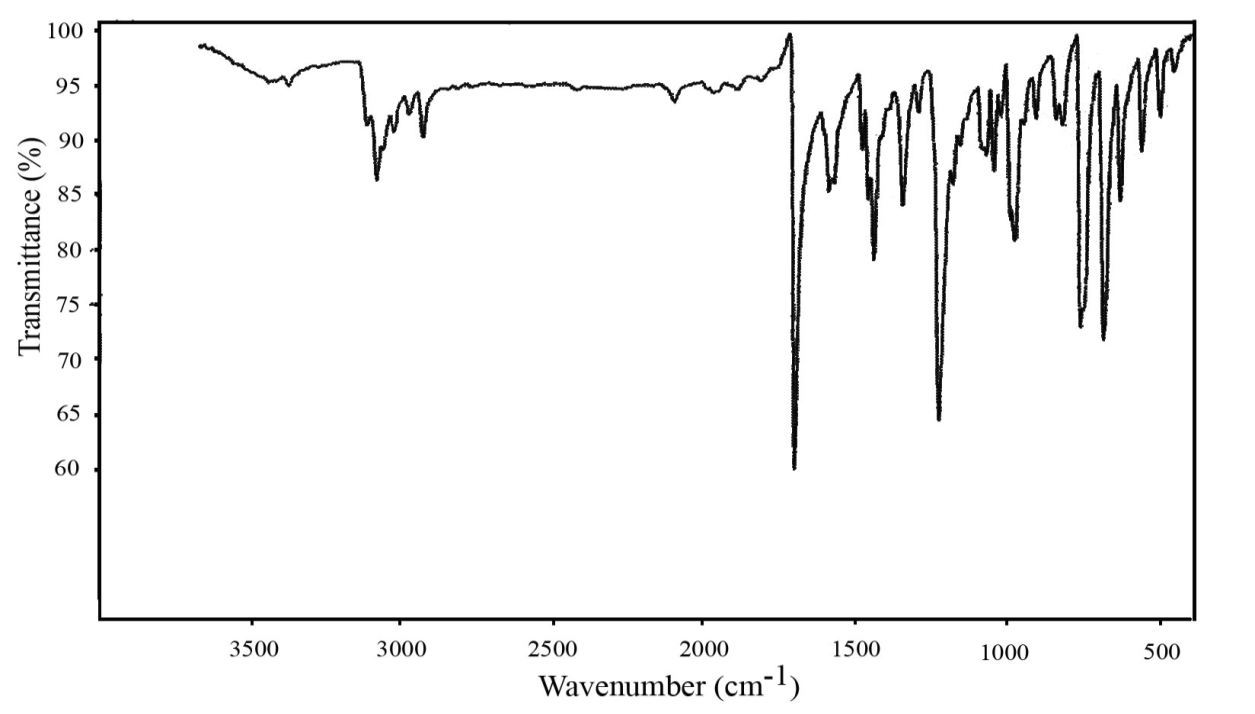


**Figure S7.** FTIR spectrum of 1-(2-bromophenyl)-2-(4-phenyl-1H-1,2,3-triazol-1-yl)ethanone


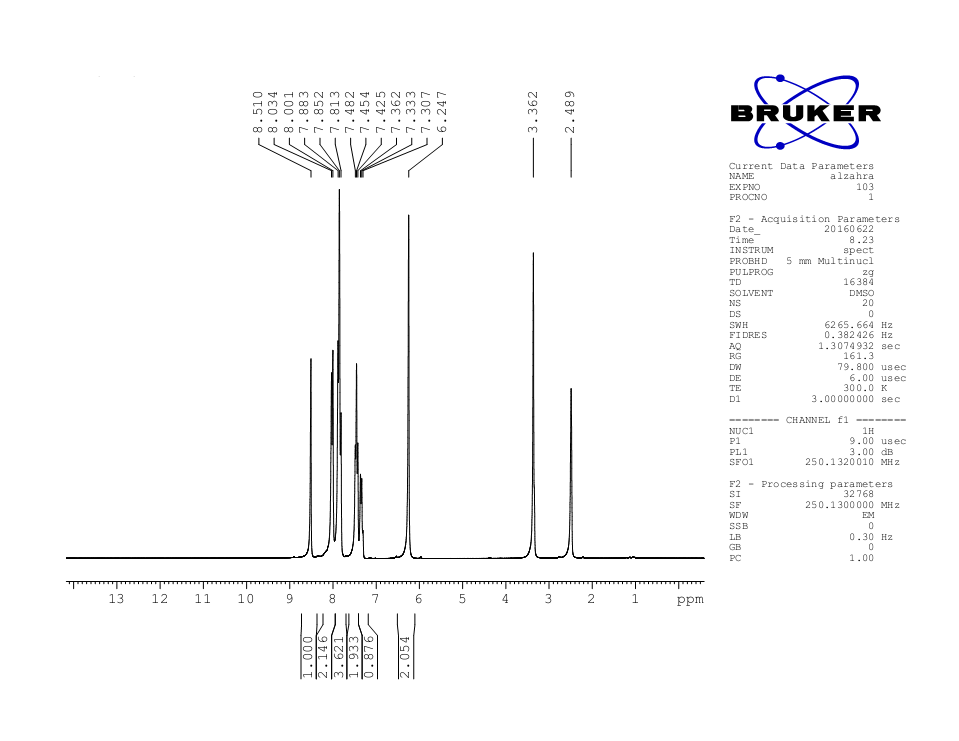
 **Figure S8.** ^1^HNMR spectrum of 1-(2-bromophenyl)-2-(4-phenyl-1H-1,2,3-triazol-1-yl)ethanone


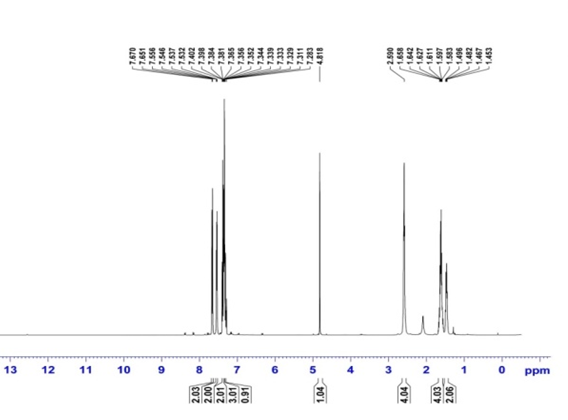


Figure S9.^1^H NMR spectrum of 1-(1-(naphthalen-3-yl)-3-phenylprop-2-ynyl)piperidine


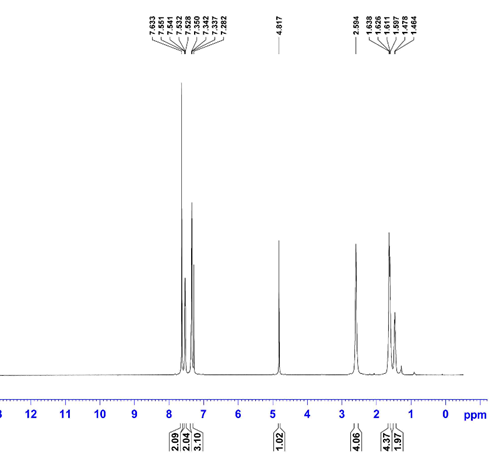


Figure S10.^1^H NMR spectrum of 1‐(3‐phenyl‐1‐(4‐(3‐phenyl‐1‐(piperidin‐1‐yl)prop‐
2‐ynyl)phenyl)prop‐2‐ynyl)piperidine
